# Reward Design for Customer Referral Programs: Reward–Product Congruence Effect and Gender Difference

**DOI:** 10.3389/fpsyg.2021.644412

**Published:** 2021-06-15

**Authors:** Hai-hua Hu, Xin-Mu Zhang

**Affiliations:** School of Management, Xi’an University of Architecture and Technology, Xi’an, China

**Keywords:** customer referral programs, referral rewards, utilitarian benefit, hedonic benefit, gender difference

## Abstract

Referral reward design is the core component of customer referral programs, which are often applied to recruit new customers. This research investigates the effectiveness of utilitarian vs. hedonic rewards in terms of referral generation. Through one field study and two laboratory studies, we demonstrate a reward–product congruency effect; that is, utilitarian rewards, compared with hedonic rewards, yield a higher referral likelihood for utilitarian products, while the opposite holds true for hedonic products. However, such a congruency effect would be crippled by gender segmentation. When males make referral decisions toward hedonic products, the effectiveness of utilitarian rewards is at least equal to that of hedonic rewards. When females make referral decisions toward utilitarian products, there is no difference in effectiveness between utilitarian and hedonic rewards. These findings provide novel insights into referral reward design.

## Introduction

Firms often offer rewards, such as cash, coupons, or gifts, to existing customers and encourage these customers to refer new customers. Such customer referral programs (CRPs) have long been considered an effective means of new customer acquisition because people often trust the referrals of friends during the buying process (Ryu and Feick, 2007; Dose et al., 2019). Unfortunately, existing customers often respond to CRPs not as actively as firms expect for a variety of reasons (Ryu and Feick, 2007; Wirtz et al., 2013). Therefore, designing an attractive referral reward is crucial for the success of CRPs (Jin and Huang, 2014; Orsingher and Wirtz, 2018).

Although specific forms are many and varied, referral rewards can be classified roughly into two types, namely, utilitarian and hedonic, in terms of the primary benefit that existing customers perceive in those rewards (Chandon et al., 2000). Consider the following examples.

- Lyft offers customers USD 10 for each referral that brought in a new user (Lyft, 2021).
- SugarSync allows current customers to get up to a maximum of 40 GB of free space, when their friend or family pays for the subscription (SugarSync, 2020).
- World of Warcraft offers users a free month of gaming if they successfully referred 10 friends to buy subscriptions (Blizzard, 2021).
- PillPack donates USD 100 to RxArt, a nonprofit organization, for every customer referral who signed up for PillPack (2020).

The USD 10 offered by Lyft and the free space offered by HomeSuite are typically utilitarian rewards, as they attract CRPs participants mainly through providing tangible, functional benefits (i.e., earning money). The 1 month of free gaming offered by World of Warcraft and the USD 100 charity donation offered by PillPack are typically hedonic rewards, which highlight the pleasure derived from the usage or possession of the rewards. The present study attempts to investigate which reward type is more effective in motivating referral generation.

Several recent studies refer indirectly to this question. For example, Jin and Huang (2014) investigated the effectiveness of monetary vs. in-kind referral rewards and showed that movie ticket vouchers (typically hedonic) can be more effective than cash (typically utilitarian). Zhang et al. (2019) addressed the analogous topic in the specific context of electronic referral and demonstrated the advantage of cinema ticket vouchers (typically hedonic) over cash. In addition, Wang et al. (2018) found that uncertain rewards generate a higher referral intention than certain rewards, because the former is with a more positive experience. However, these studies focus almost exclusively on the type of rewards and neglect other factors that may play an important role in consumers’ evaluation toward utilitarian vs. hedonic rewards.

In this study, we identified two such factors, namely, the (utilitarian/hedonic) type of the promoted product and the gender of the existing customers. Research on sales promotion states that the effectiveness of utilitarian vs. hedonic promotions is highly dependent on whether the promoted products are utilitarian or hedonic (Chandon et al., 2000; d’Astous and Landreville, 2003; d’Astous et al., 2004; Montaner et al., 2011; Buil et al., 2013). According to the benefit congruency framework (BCF), consumers prefer utilitarian (hedonic) promotions when the promoted products are utilitarian (hedonic; Chandon et al., 2000). Research on the gender difference in consumer behavior reveals that males tend to be instrumentality oriented and prefer utilitarian benefits, while females tend to be sentiment oriented and prefer hedonic benefits (Otnes and McGrath, 2001; Arnold and Reynolds, 2012; Tifferet and Herstein, 2012). We accordingly posited that product type and gender may have a critical influence on the preference of CRP participants regarding utilitarian vs. hedonic rewards.

Through one field experiment and two laboratory experiments, we demonstrated a reward–product congruency effect existing in CRPs. That is, utilitarian rewards are more effective than hedonic rewards in stimulating referral generation toward utilitarian products, and the opposite holds true in the case of hedonic products. However, this effect does not hold when males and females are segmented. Specifically, utilitarian rewards are at least not less effective than hedonic rewards in stimulating males to make referrals to hedonic products. There are no significant differences between utilitarian and hedonic rewards in terms of stimulating females to make referrals to utilitarian products. These findings are important theoretically and practically.

## Theoretical Development

### How Rewards Affect Referral Behavior

As extrinsic incentives, rewards can exert two opposing influences on referral generation. On the one hand, rewards may encourage referrals through offering economic or social benefits that compensate for the time and effort spent on making referrals (Wirtz and Chew, 2002; Ryu and Feick, 2007; Orsingher and Wirtz, 2018; Wirtz et al., 2019). As the evaluation of benefits generally is subjective and personal, the effectiveness of a reward is contingent on the perceived attractiveness of the benefits provided by the reward (Orsingher and Wirtz, 2018; Wirtz et al., 2019). Therefore, two rewards with the same face value may differ significantly in attractiveness and thus effectiveness (Jin and Huang, 2014).

On the other hand, rewards may prevent referrals with raising social impression concerns (Wirtz and Chew, 2002; Ryu and Feick, 2007; Wirtz et al., 2013, 2019; Orsingher and Wirtz, 2018). In the organic word-of-mouth context, where no reward is involved, making referrals tend to be other-oriented and altruistic (Berger, 2014). For example, people are likely to share a satisfactory restaurant with friends because they care for the wellbeing of their friends. In addition, people expect others to judge their referral behavior in the same way (Wirtz et al., 2019). However, rewards introduce an economic component into a social relationship and may therefore lead referral recipients to suspect a hidden or ulterior motivation that drives the referral behavior (Verlegh et al., 2013). To avoid such a negative impression, existing customers are less likely to engage in incentivized referrals compared with organic referrals.

Orsingher and Wirtz (2018) demonstrated that the trade-off between perceived attractiveness and social impression concerns is inherent in CRPs. It suggests that both increasing perceived attractiveness and decreasing social impression concerns can be important paths to improve referral generation. The previous literature on referral reward design focused on either or both of these paths, as discussed in the following section.

### Referral Reward Design

Related research mainly considers three factors of rewards: reward size, reward scheme, and reward type. Reward size refers to the face value (or price) of the reward (Orsingher and Wirtz, 2018). It is generally accepted in related research that a large reward has a positive effect on referral likelihood due to perceived attractiveness (Ryu and Feick, 2007; Wirtz et al., 2013, 2019; Zhang et al., 2019; Wolters et al., 2020). However, a small number of studies also show that a large reward has no effect (Kuester and Benkenstein, 2014) or even a negative effect (Wirtz et al., 2013; Jin and Huang, 2014) due to raised social impression concerns (Orsingher and Wirtz, 2018).

Reward scheme refers to those who can receive the reward (Ryu and Feick, 2007). This line of research often identifies three schemes: “reward me” (recommenders get the reward), “reward you” (recipients get the reward), and “reward both” (recommenders and recipients share the reward). “Reward both” is commonly deemed the optimal scheme because it achieves a balance between perceived attractiveness and social impression concerns (Ryu and Feick, 2007; Jin and Huang, 2014; Zhang et al., 2019; Gershon et al., 2020; Jung et al., 2021). An exception is that “reward me” becomes the most effective scheme if the relation between a recommender and a recipient is weak (Ryu and Feick, 2007). This is because consumers do not care much about how strangers would judge them and therefore can make referral decisions dependent mainly on perceived attractiveness.

Research on reward types mainly focuses on what kind of rewards can effectively reduce the social impression concerns of existing customers (e.g., Verlegh et al., 2013; Jin and Huang, 2014; Zhang et al., 2019). Cash and coupons are most frequently used in CRPs practices (Ryu and Feick, 2007); however, such monetary rewards involve a serious issue about social impression (Jin and Huang, 2014). Compared with monetary rewards, non-money rewards, including in-kind rewards (e.g., gifts) and symbolic rewards (e.g., charity donation), are deemed less likely related to social impression issues and thus more effective in recruiting CRPs participants (Verlegh et al., 2013; Jin and Huang, 2014).

The current research also investigated reward types but from another perspective: increasing perceived attractiveness. In the next section, building on BCF, we propose that existing customers may perceive a reward to be more attractive and thus show a higher referral likelihood when the reward and the promoted product belong to the same type (utilitarian or hedonic) than when they belong to different types.

### Benefit Congruency Framework

Benefit congruency framework states that the effectiveness of a sales promotion increases if its benefits provided to consumers are congruent to those of the promoted product (Chandon et al., 2000). The benefits of products and promotions are usually distinguished between utilitarian and hedonic (Chandon et al., 2000; Dhar and Wertenbroch, 2000; Okada, 2005). Utilitarian benefit is generated by the fulfillment of functional and practical needs, such as appeasing hunger, accomplishing work, or saving money. Hedonic benefit accrues through sensual and affective experiences, such as pleasure, fun, enjoyment, or fantasies. Products and promotions generally incorporate both utilitarian and hedonic benefits concurrently (Chandon et al., 2000; Voss et al., 2003). For instance, computers can be used for both work and entertainment. Despite this, past studies often categorized products and promotions into either utilitarian or hedonic, depending on the relative importance of these two types of benefits (Sinha and Verma, 2020). Simply, according to BCF, utilitarian (hedonic) promotions are more effective for utilitarian (hedonic) products.

Extant research has demonstrated BCF in various contexts of sales promotion. For example, Chandon et al. (2000) revealed that consumers prefer utilitarian monetary promotions (e.g., price reductions and coupons) if the products are utilitarian (e.g., batteries, flour, and garbage bags), but they prefer hedonic nonmonetary promotions (e.g., gifts and sweepstakes) if the products are hedonic (e.g., assorted chocolates, bubble bath, and wine). Allard et al. (2009) and Buil et al. (2013) found that consumers evaluate music downloads (hedonic gift) more positively than backpacks (utilitarian gift) when purchasing MP3 players (hedonic product), and the opposite is true when purchasing sports shoes (utilitarian product). Analogous results were also found in the contexts of purchasing computers and performing arts (d’Astous and Landreville, 2003; d’Astous et al., 2004).

There may be several psychological explanations for BCF. The first explanation is cognitive consistency (Chandon et al., 2000; Buil et al., 2013) based on congruence theory. Consumers recognize utilitarian and hedonic benefits in different ways. Utilitarian benefits are governed by functional motivation related to the tangible aspects of products and promotions, such as quality, convenience, and price. When making utilitarian decisions, therefore, consumers use a rational model. On the contrary, hedonic benefits are subjective; they are often vaguely felt or sensed by consumers through non-tangible aspects, such as store atmospherics, social status, and enjoyable experiences. In this case, consumers make decisions using an affective model. When products and promotions provide the same type of benefits, it generates cognitive consistency and further increases consumers’ evaluation and preference toward the promotions (Chandon et al., 2000).

The second explanation is the effort-congruent effect (Kivetz, 2005), which is based on a synthesis of reactance theory and overjustification theory. Consumers often react against sales promotions because they perceive promotions as “attempts to control their behavior and threats to their freedom of choice” (Kivetz, 2005). Promotions that are congruent with the consumption effort allow consumers to attribute their purchase decisions to intrinsic motivation rather than extrinsic motivation, which can reduce reactance psychology. Accordingly, under utilitarian (hedonic) consumption scenarios, consumers are less likely to react against utilitarian (hedonic) promotions than hedonic (utilitarian) ones. In addition, balance theory and categorization theory can support BCF (Czellar, 2003; Basil and Herr, 2006).

Benefit congruency framework states that consumers in a utilitarian scene value utilitarian benefits more than hedonic benefits, while those who are in a hedonic scene show the opposite tendency (Chandon et al., 2000). We accordingly posit that referral rewards offering congruent benefits with the promoted products (e.g., utilitarian product and utilitarian reward) may be more attractive for existing customers than those offering incongruent benefits with the promoted products (e.g., hedonic product and utilitarian reward). As the perceived attractiveness of rewards is a critical driver for the likelihood of existing customers to make referrals (Ryu and Feick, 2007; Orsingher and Wirtz, 2018), we propose the following hypothesis:

H1: There is a reward–product congruence effect existing in CRPs. Specifically, utilitarian rewards are more effective than hedonic rewards in stimulating referral likelihood under utilitarian product conditions, while hedonic rewards are more effective than utilitarian rewards under hedonic product conditions.

### Gender-Specific Preference for Utilitarian vs. Hedonic Benefits

It is well-documented that males and females differ inherently in terms of their preference for utilitarian vs. hedonic benefits (Wolin, 2003). Due to the evolutionary basis of competing for genetic continuity, males demonstrate competitiveness and instrumentality (Fisher, 1999); thus, they care more about functional and tangible benefits, such as performance and quality (Diep and Sweeney, 2008). By contrast, females are more relational and expressive and more likely to possess emotional mechanisms in the brain (Canli et al., 2002; Whittle et al., 2011). Therefore, females are more drawn to experiential benefits (Yang and Lee, 2010).

Such gender-specific preference (GSP; hereafter) for utilitarian vs. hedonic benefits is commendably reflected in consumer behavior. Many studies state that males see shopping as a task to fulfill instrumental needs and prefer instrumentality-oriented products, while females perceive shopping as a pleasure-seeking activity and are more attracted by items with sentimental value (e.g., Otnes and McGrath, 2001; Arnold and Reynolds, 2012; Tifferet and Herstein, 2012; Wang et al., 2021). GSP can also be observed in males’ and females’ responses to sales promotions (Harmon and Hill, 2003; Bailey, 2005; Ndubisi, 2005; Carpenter and Moore, 2008). For example, Harmon and Hill (2003) found that males are more likely than females to be heavy users of grocery store loyalty cards. Carpenter and Moore (2008) revealed that females perceive nonmonetary promotions to be more enjoyable than do males. These collectively suggest that males may respond more favorably to utilitarian referral rewards than hedonic ones, and females demonstrate the opposite.

Interestingly, BCF and GSP are coordinated in some cases but uncoordinated in other cases, as shown in Table 1. Take utilitarian products as an example; BCF and GSP consistently suggest utilitarian rewards that would be preferred by males. However, for females, BCF suggests utilitarian rewards, while GSP suggests hedonic rewards. Here, we were particularly interested in how males and females act if GSP is competing with BCF.

**Table 1:** Preferred reward type based on benefit congruency framework (BCF) and gender-specific preference (GSP).

	Utilitarian products		Hedonic products	
	BCF	GSP	BCF	GSP
Males	Utilitarian	Utilitarian	Hedonic	Utilitarian
Females	Utilitarian	Hedonic	Hedonic	Hedonic

The literature on the gender difference in information processing should be considered to answer this question. This stream of research demonstrates that males tend to be selective processors who perform item-specific processing when rendering judgment (Meyers-Levy, 1988; Meyers-Levy and Maheswaran, 1991; Putrevu, 2004). That is, they focus on a single cue that is readily available and highly salient, and they use it independently to achieve processing efficiency. It is plausible, accordingly, that males would pay close attention to benefits that are immediately offered by a reward but relatively neglect the attributes of a product that they had purchased some time ago. That is, males are influenced by GSP more than by BCF when these two mechanisms compete. This suggests that males may show a preference toward utilitarian rewards over hedonic rewards, even the products are hedonic. Considering that the effect of BCF would not collapse completely, we propose the following hypothesis:

H2: For males, utilitarian rewards are at least not less effective than hedonic rewards in stimulating referrals to hedonic products.

Empirical evidence reveals that females, on the contrary, are comprehensive processors who use relational processing (Meyers-Levy, 1988; Meyers-Levy and Maheswaran, 1991; Putrevu, 2004). When rendering judgment, females tend to assimilate all available cues received in the immediate environment and held in memory, and they elaborate the interrelationships or similarities between these cues. It implies that females are more likely than males to perceive the benefit congruency between the reward and the promoted product. In other words, females are more likely to be governed by BCF and GSP equally. It suggests that, under the utilitarian product condition, females may value utilitarian and hedonic rewards equally. Therefore, we will test the following hypothesis:

H3: For females, utilitarian and hedonic rewards have no effectiveness difference in stimulating referrals to utilitarian products.

## Overview of Studies

**Figure 1:**
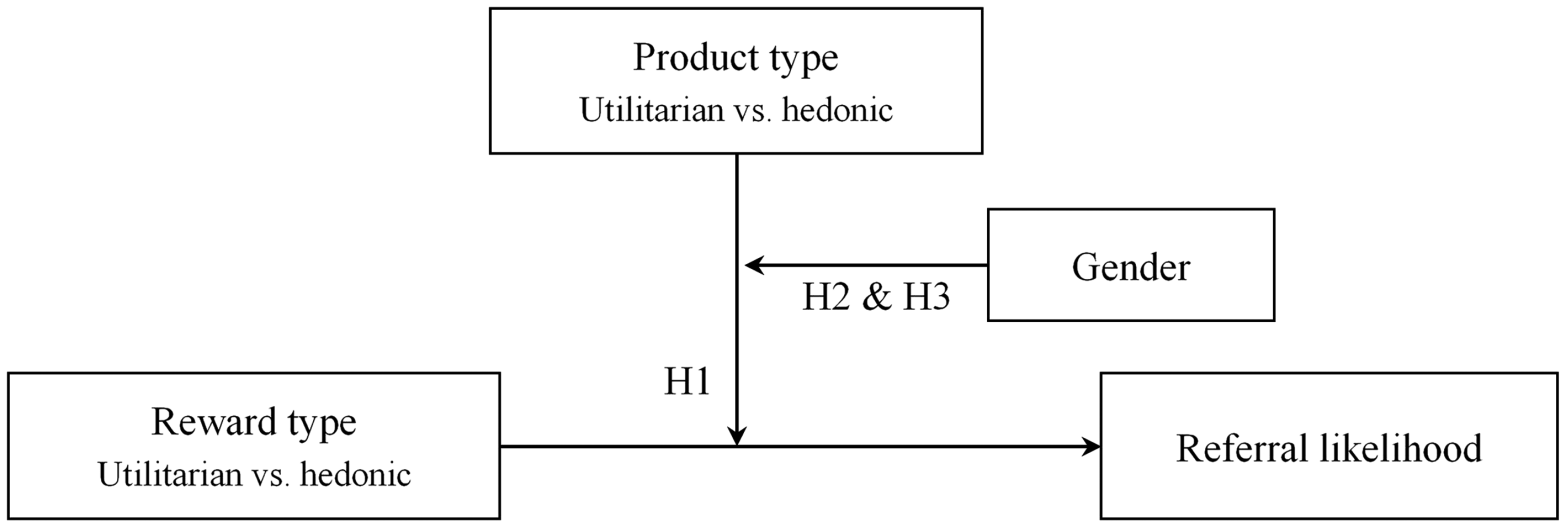
Conceptual model.

We conducted one field study and two laboratory experiments. Figure 1 illustrates our conceptual model. In Study 1, a field experiment with two Chinese companies, we tested whether the reward–product congruency effect and gender difference exist in CRPs. In this study, we used the referral conversion rate as the proxy of referral likelihood. Study 2 and Study 3 replicated the findings under different controlled conditions with varied specific forms of product and reward, reward size, and reward scheme while focusing directly on the referral likelihood.

## Study 1

### Method

#### Participants and Design

The field experiment was a 2 (reward type: utilitarian vs. hedonic) × 2 (product type: utilitarian vs. hedonic) × 2 (gender: males vs. females) between-subjects design. The first two factors (reward type and product type) were manipulated in the design. The third factor, gender, was measured afterward. The study was conducted at a fitness club and a travel agency in the main city in western China used as utilitarian and hedonic product settings, respectively. For each agency, we selected two outlets to be used as utilitarian and hedonic reward settings. The two outlets belonging to the same agency were placed at a distance of at least 15 km to void intergroup influence as much as possible. The participants were the customers of the four outlets. The sample size of the study is given in Table 2.

**Table 2:** Sample size of Study 1.

Product	Reward	Sample size		
		Males	Females	Total
Fitness membership	Fitness suit	703	533	1,236
	Relaxation massage	579	525	1,104
Outbound group tour	Travel kit	659	537	1,196
	Free excursion	631	540	1,171

#### Stimuli

After consultation with the managers of these two agencies, we chose a fitness membership (utilitarian product condition) for the fitness club and an outbound group tour (hedonic product condition) for the travel agency as product stimuli. For the fitness membership, the referral reward stimuli were set to a fitness suit (utilitarian reward condition) and relaxation massage (hedonic reward condition). For the outbound group tour, a travel kit and free excursion were used as the utilitarian and hedonic reward conditions, respectively.

#### Procedure

The experiment ran concurrently at the four outlets for 1 month starting on June 11, 2019. In the first week, over 1,100 customers selected randomly from the customer list of each outlet were informed about the CRPs through text and/or WeChat. The customers of the fitness club were told that “You will get a fitness suit, including a sports backpack, exercise towel, and one-year-free locker (or a voucher for 10-times relaxation massages after exercise), worth RMB 300 (roughly USD 43), when your friends sign up for a fitness membership for at least 1 year (one-year membership was priced at RMB 1998, roughly USD 285) through your referral.” The customers of the travel agency were told that “You will get a travel kit, including a hiking bag, sun hat, sunglasses, and towel (or a free two-day excursion), worth RMB 350 (roughly USD 50), when your friends sign up for an outbound group tour above RMB 5000 (roughly USD 710) through your referral.”

Considering that the participants would receive the rewards only for successful referrals, we did not ask for feedback on whether they sent invitations to their friends. When the referred customers signed contracts, we asked them to provide the name and contact information of their referrers. Therefore, we had data on the referral conversions but not the actual referrals (including successful and failed referrals). Related research has proven that the rewarded referral rate is closely related to the conversion rate (Gershon et al., 2020; Jung et al., 2020). Moreover, since the rewards were offered to recommenders only, the differences in reward type should theoretically have no effect on the purchase decision of the referral recipients. For the above reasons, we used the conversion rate as a proxy for the referral likelihood.

### Results

#### Manipulation Checks

A pretest was conducted to check the manipulation of the product type and reward type, which involved the same populations as those of the main study (*N =* 36 from the fitness club and *N =* 40 from the travel agency). We first showcased the participants of each agency with one product and two rewards correspondingly. The participants then were asked to indicate how utilitarian/hedonic they perceived these three items to be based on a 6-point scale (1 = “very utilitarian” and 6 = “very hedonic”). We did not expose the actual application of the rewards to avoid interfering with the participants’ perception. Following Khan and Dhar (2010), we defined the items scored below three as utilitarian and items scored over four as hedonic. Results showed that the participants can accurately identify the utilitarian type regarding the fitness membership (*M* = 2.50) and the hedonic type regarding the outbound group travel [*M* = 4.78; *t*(74) = 9.19, *p* < 0.001]. The participants from the fitness club classified the fitness suit (*M* = 2.22) as utilitarian while the relaxation massage as hedonic [*M* = 4.47; *t*(70) = 7.44, *p* < 0.001]. The participants from the travel agency categorized the travel kit (*M* = 2.48) as utilitarian while the excursion as hedonic [*M* = 4.68; *t*(78) = 7.12, *p* < 0.001]. These results validated our manipulation.

#### Reward–Product Congruency Effect Test

**Figure 2:**
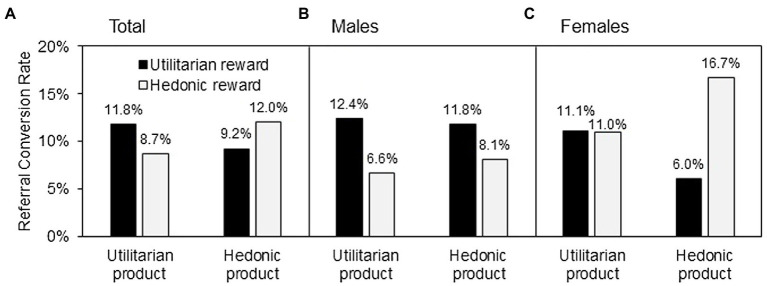
Study 1 results.

A binary logistic regression was conducted, with the congruency between product and reward (1 for congruency and 0 for incongruency), product type, and gender as independent variables and conversion as the dependent variable, to test H1. Multiple successful referrals made by one existing customer were counted only once, because the focus of this study is the referral generation. The results indicated the reward–product congruency effect [*χ*^2^(1) = 7.60, *p* < 0.01], as expected by H1 and presented in Figure 2A. Product type [*χ*^2^(1) = 0.71, ns] and gender [*χ*^2^(1) = 0.01, ns] did not influence the results. Under utilitarian product condition, the conversion rate was higher when the reward was utilitarian (11.8%) than when it was hedonic [8.7%; *χ*^2^(1) = 6.31, *p* < 0.05]. Under hedonic product condition, the conversion rate was higher when the reward was hedonic (12.0%) than when it was utilitarian [9.2%; *χ*^2^(1) = 4.97, *p* < 0.05].

#### Gender Difference Test

We added the interaction of congruency and gender to the binary logistic regression model, showing that gender [*χ*^2^(1) = 5.23, *p* < 0.05] significantly moderated the effect of congruency on the conversion rate. A further analysis showed that, for the male participants, the utilitarian rewards always produced a higher conversion rate than did the hedonic rewards, regardless of whether the product was utilitarian [12.4% vs. 6.6%; *χ*^2^(1) = 11.82, *p* < 0.005] or hedonic [11.8% vs. 8.1%; *χ*^2^(1) = 4.99, *p* < 0.05], as illustrated in Figure 2B. For the female participants, the hedonic rewards produced a higher conversion rate than did the utilitarian rewards when the product was hedonic [16.7% vs. 6.0%; *χ*^2^(1) = 28.37, *p* < 0.001]. Meanwhile, the effects of the utilitarian and hedonic rewards on the conversion rate were insignificant when the product was utilitarian [11.1% vs. 11.0%; *χ*^2^(1) = 0.01, ns], as presented in Figure 2C. These results supported H2 and H3.

### Discussion

Using the customers of the two agencies, Study 1 showed that the congruency between products and rewards was beneficial to the CRPs. However, such an effect did not hold when genders were distinguished. Specifically, males always preferred utilitarian rewards, while females preferred hedonic rewards under hedonic product condition but treat hedonic and utilitarian rewards equally under utilitarian product condition. These results supported H1–H3. In this field study, we could not establish appropriate controls for the price of the promoted product and reward as well as their price ratio. Moreover, we employed the conversion rate as a proxy for the referral likelihood. In the two following laboratory experiments, we attempted to replicate Study 1’s results under more rigorous controls while measuring the referral likelihood directly.

## Study 2

### Method

#### Participants and Design

Study 2 employed a 2 (reward type: utilitarian vs. hedonic) × 2 (product type: utilitarian vs. hedonic) × 2 (gender: males vs. females) between-subjects design. The first two factors were manipulated, while the third factor was measured at the end of the questionnaire. A total of 284 undergraduates (140 females) at a university in western China were randomly assigned to one of the eight conditions.

#### Stimuli

In this study, the promoted products included a mini-type washer (utilitarian) and one-day funfair pass card (hedonic), both of which were priced at RMB 299 (roughly USD 43). The referral rewards were one 2 kg bag of liquid detergent (utilitarian) or two movie tickets (hedonic), both of which were priced at RMB 50 (roughly USD 7, 16.7% of the price of the promoted product).

#### Procedure

We first asked the participants to read the specifications of the promoted product. A picture showing the product with a price tag and usage scenario was also provided to reinforce the authenticity of the simulation. To avoid the influence of prior brand beliefs, we used a fictional brand name. In accordance with the convention of CRP research (Ryu and Feick, 2007; Jin and Huang, 2014), after reading the material, the participants were asked to imagine that they had bought and used/experienced the product and were very satisfied with it.

Next, the participants were informed that the firm was conducting a CRP in which customers would be offered a reward for recommending the product to someone who then purchases the same model. After learning about the CRP, the participants indicated their referral likelihood on an 11-point rating scale (0 = “will not recommend with certainty” and 10 = “will recommend with certainty”). Afterward, the participants were asked to indicate the product type and reward type (1 = “very utilitarian” and 6 = “very hedonic”). Finally, demographic information, including gender, age, and monthly consumption, was collected to control for individual differences.

### Results

#### Manipulation Checks

The participants categorized the mini-type washer (*M* = 2.27) and funfair pass card [*M* = 4.50; *t*(282) = 9.02, *p* < 0.001] as utilitarian and hedonic, respectively. They classified the liquid detergent (*M* = 2.74) and movie tickets [*M* = 4.97; *t*(282) = 7.45, *p* < 0.001] as utilitarian and hedonic, respectively. These results revealed that our manipulations were successful.

#### Reward–Product Congruency Effect Test

**Figure 3:**
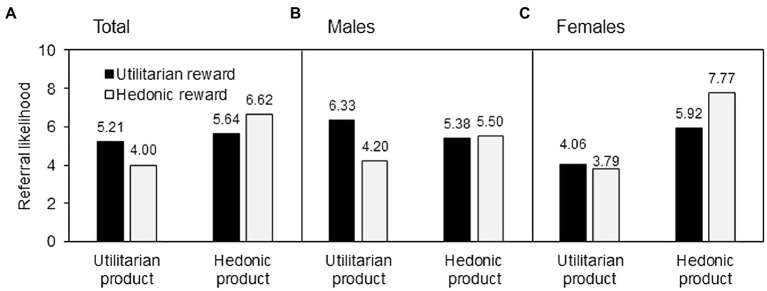
Study 2 results.

An initial two-way between-subjects ANOVA was conducted to test H1. The main effect of product type [*F*(1, 280) = 20.67, *p* < 0.001] was significant, and that of reward type [*F*(1, 280) = 0.98, ns] was insignificant. The ANOVA showed an interaction between the product type and the reward type [*F*(1, 280) = 10.61, *p* < 0.005], as depicted in Figure 3A. A simple effect analysis showed that, for the utilitarian products, the participants under the utilitarian reward condition (*M* = 5.21) indicated a higher referral likelihood than those under the hedonic reward condition [*M* = 4.00; *F*(1, 280) = 6.42, *p* < 0.05]. For the hedonic products, the participants under the hedonic reward condition (*M* = 6.62) indicated a higher referral likelihood than those under the utilitarian reward condition [*M* = 5.64; *F*(1, 280) = 4.29, *p* < 0.05]. These results demonstrated the reward–product congruency effect that was proposed in H1.

#### Gender Difference Test

A three-way between-subjects ANOVA was conducted to test H2 and H3. The main effect of product type [*F*(1, 276) = 22.84, *p* < 0.001] was significant, while those of reward type [*F*(1, 276) = 0.11, ns] and gender [*F*(1, 276) = 0.01, ns] were insignificant. The effects of product type × reward type (*F*(1, 276) = 11.43, *p* < 0.005), product type × gender [*F*(1, 276) = 18.03, *p* < 0.001], and reward type × gender [*F*(1, 276) = 7.76, *p* < 0.01] were significant. The interaction of product type, reward type, and gender had no effect on the referral likelihood [*F*(1, 276) = 0.01, ns]. Under the utilitarian product condition, the male participants (Figure 3B) reported a higher referral likelihood when the reward was utilitarian (*M* = 6.33) than when the reward was hedonic [*M* = 4.20; *F*(1, 276) = 10.89, *p* < 0.005], while the female participants (Figure 3C) reported no difference in referral likelihood between the utilitarian (*M* = 4.06) and hedonic rewards [*M* = 3.79; *F*(1, 276) = 0.61, ns]. Under the hedonic product condition, the female participants (Figure 3C) reported a higher referral likelihood when the reward was hedonic (*M* = 7.77) than when the reward was utilitarian [*M* = 5.92; *F*(1, 276) = 8.23, *p* < 0.005], while the male participants (Figure 3B) reported no difference in referral likelihood between the utilitarian (*M* = 5.38) and hedonic rewards [*M* = 5.50; *F*(1, 276) = 0.04, ns]. These results supported H2 and H3.

### Discussion

Study 2 focused directly on the referral likelihood. It again demonstrated the reward–product congruency effect (H1) and gender difference (H2 and H3) in CRPs. It needs to be noted, nevertheless, that Study 2 did not replicate the results of Study 1 completely. Specifically, for hedonic products, males showed a preference for utilitarian rewards over hedonic rewards in Study 1 but indicated no preference in Study 2. This can be explained by the fact that the cue about the promoted product is less salient, and thus, GSP exerts a greater effect in the field study setting than in the laboratory study setting.

In Study 2, the simulated CRPs were designed with relatively low-priced products and rewards as well as the “reward me” scheme. Related research reveals that these factors can exert a critical influence on referral likelihood (Ryu and Feick, 2007; Jin and Huang, 2014; Orsingher and Wirtz, 2018). In the following laboratory study, we duplicated the experiment with relatively high-priced products and rewards as well as the “reward both” scheme to verify our previous conclusions. Some scholars suggest that BCF may be a misconception of the complementary relationship between promoted products and their promotions (e.g., mini-type washer and liquid detergent used in Study 2; Kim and Min, 2016). In Study 3, we chose rewards whose complementarity with the selected products was as small as possible.

## Study 3

### Method

#### Participants and Design

Study 3 used a 2 (reward type: utilitarian vs. hedonic) × 2 (product type: utilitarian vs. hedonic) × 2 (gender: males vs. females) between-subjects design. Gender was determined at the end of the questionnaire. A total of 296 MBA students (148 females) at the same university of Study 2 participated in this study for partial course credit and were randomly assigned to one of the eight conditions.

#### Stimuli

A one-week course of leadership development and a five-day wild adventure were selected as utilitarian and hedonic products, respectively. Both of them were priced at RMB 5999 (roughly USD 857). Cash worth RMB 600 (roughly USD 86, 10% of the price of the promoted product) was used as the utilitarian reward. The hedonic reward was RMB 600 worth of vouchers that could be redeemed for two concert tickets.

#### Procedure

The procedure was the same as that of Study 2. The participants were first guided to read the material of the promoted product and image product satisfaction. Then, they were informed about the CRPs. Unlike in Study 2, the reward in this study was divided equally between the referrer and the referee. Afterward, the participants scored their referral likelihood on a 101-point rating scale (0 = “will not recommend with certainty” and 100 = “will recommend with certainty”). The manipulation check included product type and reward type (1 = “very utilitarian” and 6 = “very hedonic”). Demographic information, including gender, was collected at the end of the questionnaire.

### Results

#### Manipulation Checks

The participants affirmed the utilitarian and hedonic nature of the leadership development course (*M* = 2.57) and wild adventure [*M* = 5.27; *t*(294) = 11.34, *p* < 0.001], respectively, and perceived no difference in pricing reasonableness between them [*M* = 5.57 vs. *M* = 5.36; *t*(294) = 1.16, ns]. The cash was classified as a utilitarian reward (*M* = 2.85), while the concert ticket voucher was attributed to hedonic reward [*M* = 5.11; *t*(294) = 9.84, *p* < 0.001] These results showed that our manipulations were successful.

#### Reward–Product Congruency Effect Test

**Figure 4:**
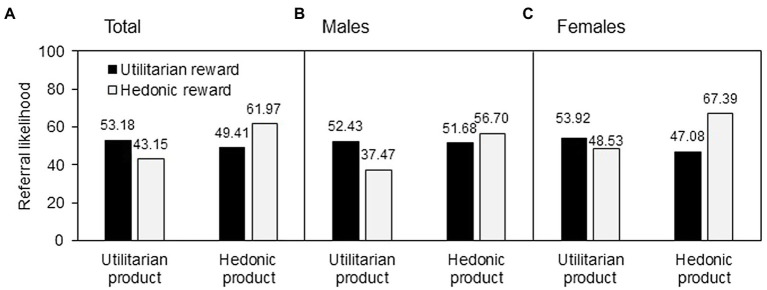
Study 3 results.

We first conducted a two-way between-subjects ANOVA to test H1. The main effect of product type [*F*(1, 292) = 5.61, *p* < 0.05] on the referral likelihood was significant, but that of reward type [*F*(1, 292) = 0.16, ns] was insignificant. The product type × reward type interaction was significant [*F*(1, 292) = 12.61, *p* < 0.001], as illustrated in Figure 4A. Under the utilitarian product condition, the utilitarian reward (*M* = 53.18) induced a higher referral likelihood than did the hedonic reward [*M* = 43.15; *F*(1, 280) = 4.97, *p* < 0.05]. Under the hedonic product condition, the hedonic reward (*M* = 49.41) induced a higher referral likelihood than did the utilitarian reward [*M* = 61.97; *F*(1, 280) = 7.80, *p* < 0.01]. These results demonstrated the reward–product congruency effect, supporting H1.

#### Gender Difference Test

A three-way between-subjects ANOVA was performed to test H2 and H3. The main effect of product type [*F*(1, 288) = 5.80, *p* < 0.05] was significant, while those of reward type [*F*(1, 288) = 0.15, ns] and gender [*F*(1, 288) = 2.16, ns] were insignificant. The effect of product type × reward type was significant [*F*(1, 288) = 12.99, *p* < 0.001]; that of product type × gender was insignificant [*F*(1, 288) = 0.26, ns]; and that of reward type × gender was marginally significant [*F*(1, 288) = 3.85, *p* < 0.1]. The interaction of product type, reward type, and gender had no effect on the referral likelihood [*F*(1, 288) = 0.20, ns]. For male participants (Figure 4B), the utilitarian reward induced a higher likelihood than did the hedonic reward under the utilitarian product condition [*M* = 52.43 vs. *M* = 37.47; *F*(1, 288) = 5.50, *p* < 0.05], but they had no difference under the hedonic product condition [*M* = 51.68 vs. *M* = 56.70; *F*(1, 288) = 0.64, ns]. For female participants (Figure 4C), the hedonic reward induced a higher likelihood than did the utilitarian reward under the hedonic product condition [*M* = 67.39 vs. *M* = 47.08; *F*(1, 288) = 10.14, *p* < 0.005], while no difference existed between utilitarian and hedonic reward under the utilitarian product condition [*M* = 53.92 vs. *M* = 48.53; *F*(1, 288) = 0.73, ns]. These results supported H2 and H3.

### Discussion

Study 3 confirmed that the reward–product congruency effect and gender difference were robust for the various settings of the reward size (whether measured by the reward’s absolute price or by the percent of the product price) and reward scheme. Moreover, Study 3’s results are important theoretically in that they show that the reward–product congruency effect comes not out of the complementarity between product and reward, when cash was used as the utilitarian reward.

## General Discussion

Marketers struggle to design effective and profitable incentives that encourage existing customers to refer new customers. The current research examines the effects of utilitarian vs. hedonic referral rewards on referral generation. One field experiment and two laboratory experiments provide sufficient evidence for the reward–product congruency effect; that is, utilitarian rewards, compared with hedonic rewards, can induce a higher referral likelihood toward utilitarian products, while the opposite holds toward hedonic products. Our research also reveals that the reward–product congruency effect could be crippled by gender segmentation. When males make referral decisions toward hedonic products, the effectiveness of utilitarian rewards becomes at least equal to that of hedonic rewards. When females make referral decisions toward utilitarian products, the effectiveness of utilitarian and hedonic rewards does not differ.

### Theoretical Contributions

This research, at the most basic level, contributes to the CRP literature by identifying referral rewards based on their utilitarian/hedonic attribute. Prior research focused mainly on the effects of reward size and reward scheme on rewarded referral likelihood (e.g., Ryu and Feick, 2007; Verlegh et al., 2013; Wirtz et al., 2013, 2019; Orsingher and Wirtz, 2018). Only a few studies explored reward type (e.g., Jin and Huang, 2014; Wang et al., 2018; Zhang et al., 2019); nevertheless, they emphasize the explicit forms (e.g., monetary vs. in-kind) but overlook the implicit nature (utilitarian or hedonic) of the rewards. Our findings reveal that the effectiveness of utilitarian and hedonic rewards, even with the same explicit form (e.g., both liquid detergent and movie tickets used in Study 2 are in-kind), can be considerably diverse.

Our findings add to the BCF literature from three aspects. First, the findings of the reward–product congruence effect extend BCF to a context in which people are incentivized to share word-of-mouth with their friends. Although BCF has been tested in a variety of contexts, the previous research focused on incentivizing people to make purchases that have consequences for only themselves (e.g., Chandon et al., 2000; d’Astous and Landreville, 2003; d’Astous et al., 2004; Montaner et al., 2011; Buil et al., 2013). This research demonstrates that BCF can apply to the context of social interactions (i.e., CRPs) as well. Second, the results of Study 3 clarify that BCF is not a misconception of the complementarity between products and promotions. Third, the results about gender difference reveal that the effect of BCF would decrease as gender segmentation, due to the distinctive, inherent preferences of males and females for utilitarian vs. hedonic benefits.

Our findings also add to the empirical evidence on gender difference in the CRP context. Gender difference has been extensively documented in the marketing literature (Harmon and Hill, 2003; Bailey, 2005; Ndubisi, 2005; Carpenter and Moore, 2008); however, it did not receive sufficient attentions in studies focused on CRPs. Our findings indicate that the effectiveness of utilitarian vs. hedonic rewards in stimulating referrals differs between males and females. This calls for careful consideration of the effect of sample structure when studying CRPs.

### Managerial Implications

Our findings suggest two major managerial implications. First, firms can improve CRP performance by matching referral rewards to the promoted product according to their utilitarian/hedonic attribute. Simply, a utilitarian (hedonic) reward, regardless of its specific form, should be selected for a utilitarian (hedonic) product. For example, the retailers, e.g., selling household goods, health foods, or financial products, could offer customers a useful reward, such as cash, coupon, rebate, gift card, or practical gift. Travel agencies and online game providers, on the contrary, could offer customers an emotional reward, such as toys, movie tickets, or exclusive fragrance. The significant benefit of this strategy is that it can effectively increase customer referrals at little extra cost.

Second, firms can further improve CRP performance by adjusting the reward type according to gender segmentation. If customers are mainly males, utilitarian rewards are a better option for CPRs; otherwise, hedonic rewards are a safer choice. If the customer base is mixed-gender, marketers can prepare two options and offer the utilitarian one to males while the hedonic one to females.

There is a derivative implication that marketers need to have a clear understanding of how CRP targets perceive the offered rewards and the promoted product. A specific reward or product generally incorporates both utilitarian and hedonic benefits concurrently (Chandon et al., 2000; Voss et al., 2003), so that the perceived type of which may significantly differ across customer segmentations. For example, tablets are working equipment for some consumers but entertainment equipment for others. Such a perception difference in utilitarian/hedonic type is especially easy to appear between males and females (Wolin, 2003). Therefore, the misjudgment of reward type and product type may decrease CRP performance.

### Limitations and Further Research

Despite the implications of our findings, this research has limitations that offer opportunities for further research. First, we concentrated on the effect of reward type interacting with product type and gender on the referral likelihood. Evidence indicates other factors, such as brand strength and the quality of the recommender–receiver relation, that are influential for rewarded referrals. For example, Ryu and Feick (2007) found that people respond differently to referral rewards depending on whether they recommend a strong or weak brand or whether they make referrals to close friends or acquaintances. It would be fruitful to incorporate these contextual factors into future research.

Second, we did not investigate how personal background variables might affect people’s evaluation and preference toward utilitarian/hedonic rewards. For example, evidence shows that, when shopping, low-income consumers are more attracted by hedonic benefits, while high-income consumers tend to be appealed by utilitarian benefits (Allard et al., 2009). These issues await future investigation.

Finally, the results of these three studies rely on a sample of Chinese participants. There is evidence that Chinese consumers have different orientations of utilitarian vs. hedonic consumption compared with those who have other cultural backgrounds (Lim and Ang, 2008). Therefore, our findings should be generalized with caution, and additional research might be needed to strengthen the validity of the above-reported results for other cultural contexts.

## Data Availability Statement

The raw data supporting the conclusions of this article will be made available by the authors, without undue reservation.

## Ethics Statement

Ethical review and approval were not required for the study on human participants in accordance with the local legislation and institutional requirements. Written informed consent for participation was not required for this study in accordance with the national legislation and the institutional requirements.

## Author Contributions

H-hH contributed to put forward ideas, building models, and writing the manuscript. X-MZ contributed to experimental design and implementation, collect the data, and data analysis. All authors contributed to the article and approved the submitted version.

### Conflict of Interest

The authors declare that the research was conducted in the absence of any commercial or financial relationships that could be construed as a potential conflict of interest.
